# Which Patients Do We Need to Test for BRCA1/2 Mutation? Feasibility of Adjuvant Olaparib Treatment in Early Breast Cancer–Real-World Data from Two Large German Breast Centers

**DOI:** 10.3390/cancers15153847

**Published:** 2023-07-28

**Authors:** Dominik Dannehl, Tobias Engler, Léa Louise Volmer, Christian Martin Tegeler, Julia Fusshoeller, Emma Gabrysch, Kenneth Eissler, Anna Seller, Eva-Maria Grischke, Markus Hahn, Ines Gruber, Fabienne Schochter, Kerstin Pfister, Kristina Veselinovic, Elena Leinert, Brigitte Rack, Visnja Fink, Wolfgang Janni, Sara Yvonne Brucker, Andreas Daniel Hartkopf, Henning Schäffler

**Affiliations:** 1 Department of Women’s Health, Tuebingen University, 72076 Tuebingen, Germany; 2 Department of Gynecology and Obstetrics, University Hospital, 89075 Ulm, Germanyhenning.schaeffler@uniklinik-ulm.de (H.S.)

**Keywords:** breast cancer, BRCA, olaparib

## Abstract

**Simple Summary:**

Targeted therapies are increasingly used in patients with early breast cancer and a high clinical risk of relapse. Patients with clinical high-risk HER2-negative early breast cancer might be eligible for olaparib treatment. A prerequisite for olaparib treatment is a germline BRCA1/2 mutation. In the clinical routine, patients undergo BRCA1/2 mutation testing if they show family breast or ovarian cancer history. However, it is not known whether all patients that are potentially eligible for olaparib treatment are recommended to undergo BRCA1/2 mutation assessment based on family history. Thus, the aim of this study was to analyze which patients are eligible for olaparib treatment based on the OlympiA trial and to clarify which of these patients do not undergo BRCA1/2 mutation testing in the clinical routine.

**Abstract:**

Background: Approximately 6% of women with breast cancer carry pathogenic germline variants in predisposition genes such as BRCA1 and BRCA2. Depending on personal and family cancer history, it is therefore recommended to test for hereditary breast cancer. Moreover, as shown by the phase III OlympiA trial, olaparib significantly improves overall survival in patients with HER2 negative (HER2−) early breast cancer who (1) carry a BRCA1 or BRCA2 germline mutation (gBRCA1/2-positive), (2) have received (neo)adjuvant chemotherapy and (3) are at high clinical risk. The objective of the current analysis was to determine the number of patients with early HER2− breast cancer who are at high clinical risk, according to the inclusion criteria of OlympiA, and to estimate how many of these patients would meet the criteria for hereditary cancer testing in a real-world analysis. Methods: All patients included in this retrospective analysis were treated for early breast cancer (eBC) at the Department of Gynecology and Obstetrics, Ulm University Hospital, Germany, and the Department of Women’s Health at Tuebingen University Hospital, Germany, between January 2018 and December 2020. Patients were identified as high risk, in line with the clinicopathological determiners used in the OlympiA trial. The criteria of the German Consortium for Hereditary Breast and Ovarian Cancer were used to identify patients who qualify for hereditary cancer testing. Results: Of 2384 eligible patients, 1738 patients (72.9%) showed a hormone receptor positive (HR+)/HER2− tumor biology, 345 patients (14.5%) displayed HER2+ breast cancer and 301 patients (12.6%) suffered from HR-/HER2− breast cancer (TNBC). Of 2039 HER2− breast cancer patients, 271 patients (13.3%) were at high clinical risk. This cohort encompassed 130 of the 1738 patients with HR+/HER2− breast cancer (7.5%) and 141 of 301 patients with TNBC (46.8%). A total of 121 of 271 patients (44.6%) with high clinical risk met the criteria for hereditary cancer testing (34 of 130 (26.2%) HR+/HER2− patients and 87 of 141 (61.7%) patients with TNBC). Conclusion: Approximately one in ten patients with HR+/HER2−, and half of the patients with TNBC, meet the high-risk criteria according to OlympiA. Half of these patients do not meet the criteria for hereditary cancer testing and should therefore be tested for the presence of gBRCA1/2 mutations, irrespective of their own or family cancer history. The overall number of patients with early breast cancer benefiting from olaparib needs to be investigated in future studies.

## 1. Introduction

Treatment of late-stage breast cancer has been significantly improved by the emergence of personalized approaches that have extended patients’ lives. These treatment options are now being used in earlier, curative therapy settings to prevent the emergence of distant metastatic disease. Although neoadjuvant or adjuvant chemotherapy is still the standard of care for patients with high-risk early breast cancer, several additional oral treatment options like abemaciclib, neratinib and olaparib are currently available [1,2,3,4]. As shown in the randomized phase III OlympiA trial, the poly ADP ribose polymerase (PARP) inhibitor olaparib is effective in patients with HER2-negative (HER2−) early breast cancer (eBC) who carry germline BRCA1 and BRCA2 mutations in the susceptibility genes BRCA1 and BRCA2 (gBRCA1/2 positive), that have received neoadjuvant or adjuvant chemotherapy and are at clinical high risk of relapse [5,6]. The intake of 300 mg olaparib twice daily significantly improved overall survival, with a 3.7% reduction in the risk of death after three years, alongside a hazard ratio of 0.68 and a 99% confidence interval of 0.44–1.05 [5]. These results led to the approval of olaparib by the European Medicines Agency for patients with gBRCA1/2-mutated high-risk hormonal receptor positive (HR+)/HER2− eBC or triple-negative early breast cancer (TNBC). To estimate the proportion of patients with eBC who need to undergo gBRCA1/2 testing for olaparib treatment, irrespective of their own and family cancer history, the aim of this study was to analyze how many patients fulfill the inclusion criteria of the OlympiA trial and to estimate how many of these patients meet the criteria for hereditary cancer testing in a real-world analysis.

## 2. Material and Methods

All patients included in this retrospective analysis were treated for eBC at the Department of Gynecology and Obstetrics, Ulm University Hospital, and the Department of Women’s Health at Tuebingen University Hospital in Germany between January 2018 and December 2020. This study was conducted according to the guidelines of the Declaration of Helsinki and approved by the Ethics Committee of both Tuebingen University Hospital (protocol code 379/2022BO2) and Ulm University Hospital (protocol code 72/23-FSt./bal.). Patients (female and male) were eligible for this retrospective analysis if they underwent complete surgical resection (R0). If patients were diagnosed with bilateral breast cancer, the tumor with the worse prognosis was included in the analysis. Hormone receptor (HR) and HER2 receptor expression were assessed by board-certified pathologists according to local standards: tumors were defined as HR+ if they had a positive estrogen receptor (ER) and/or a positive progesterone receptor (PR) expression according to immunohistochemistry (≥10% for ER, ≥10% for PR). HER2 immunoreactivity was scored on a scale of 0 to 3+ using the HERCEPT test (DAKO, Glostrup, Denmark). Only tumors with a HER2 score of 3+ or 2+ with detectable HER2 amplification were counted as HER2 positive. Tumors with a HER2 score of 1+ or 2+ without detectable HER2 amplification were classified as HER2 low. If no HER2 immunoreactivity could be found (score of 0), tumors were classified as HER2 0. As a simplified representation, HER2 low and HER2 negative receptor status was summarized as HER2 negative. HER2 amplification was determined by fluorescence in situ hybridization using the Pathvysion^®^ Kit (Vysis, Downers Grove, IL, USA) in Tuebingen and the ZytoMation^®^ ERBB2/CEN 17 Dual Color FISH Probe (Cytovision GmbH, Bremerhaven, Germany) in Ulm. The CPS-EG score was assessed according to the pre-therapeutic clinical stage (CS) and the post therapeutic pathological stage (PS), as well as the estrogen receptor expression (E) and the grading (G) in the neoadjuvant therapy setting as previously described [7].

According to the inclusion criteria of OlympiA, clinical high risk was defined as follows [5]: Patients with TNBC treated with neoadjuvant chemotherapy had to display residual invasive tumor in the histopathological analysis (i.e., no pathological complete response, pCR). For patients with TNBC who did not receive neoadjuvant chemotherapy, either pathologic lymph node involvement or a tumor size of at least 20 mm had to be present. Patients with HR+/HER2− eBC who underwent neoadjuvant chemotherapy also had to display non-pCR in the histopathological analysis. Additionally, they needed to exhibit a CPS-EG score of at least three. Patients with HR+/HER2− breast cancer who did not receive neoadjuvant chemotherapy had to have at least four pathologically involved lymph nodes.

Patients who qualify for hereditary cancer testing were determined using the criteria of the German Consortium for Hereditary Breast and Ovarian Cancer (GC-HBOC) (Table S2) [8,9,10,11].

Data processing and statistical analyses were performed using Jupyter Notebook (Version 6.3.0, Project Jupyter, open-access and community developed) on Anaconda (Version 3.0, Anaconda Inc., Austin, TX, USA), with the Python extension packages pandas (Version 1.4.1, open-access and community developed) and numeric Python (Version 1.22.2, open-access and community developed). Wondershare EdrawMind (Wondershare Technology Co. Ltd. Shenzhen, Guangdong, China) was used for designing flow charts and data visualization.

## 3. Results

In total, 2384 patients with eBC were included in this retrospective analysis. The most common tumor subtype was HR+/HER2− (72.9%), followed by HER2+ (14.5%) and TNBC (12.6%). Since only patients with HER2− eBC were included in the OlympiA study, the following section will focus on these patients only. Patient characteristics of the whole study population can be reviewed in Supplementary Table S1.

A total of 1738 patients had an HR+/HER2− tumor, and 301 patients had a triple-negative tumor biology (Table 1). The average age of patients with HR+/HER2− eBC was 60.1 ± 12.3 years old. Most patients with HR+/HER2− eBC were postmenopausal (1203/1738; 69.2%), the most common histology was non-special type (NST; 1360/1738; 78.2%), and the most common grading was G2 (1247/1738; 71.8%). Most patients with HR+/HER2− eBC had a tumor smaller than 20 mm (1084/1738; 62.3%) and did not display pathologically involved lymph nodes (1195/1738; 68.8%). Most patients with HR+/HER2− eBC displayed HER2 low receptor status (1118/1738; 64.3%). Furthermore, most patients with HR+/HER2− eBC did not receive (neo)adjuvant chemotherapy (1279/1738; 73.6%). Patients with TNBC were on average 55.6 ± 14.9 years old, and approximately half of the patients were postmenopausal (162/301; 53.8%). The most common histology in patients with TNBC was NST (275/301; 91.4%), and the most common grading was G3 (238/301; 79.1%). After surgery, 77.8% (234/301) of patients with TNBC who either received neoadjuvant chemotherapy or primary surgery displayed a tumor size smaller than 20 mm, and 83.7% (288/301) had no pathologically involved lymph nodes. Most patients with TNBC displayed HER2 0 receptor status (182/301; 60.5%). The majority of patients with TNBC received neoadjuvant chemotherapy (188/301; 62.4%), 72/301 (23.9%) received adjuvant chemotherapy and only 13.6% (41/301) of the patients with TNBC did not receive chemotherapy at all.

**Figure 1:**
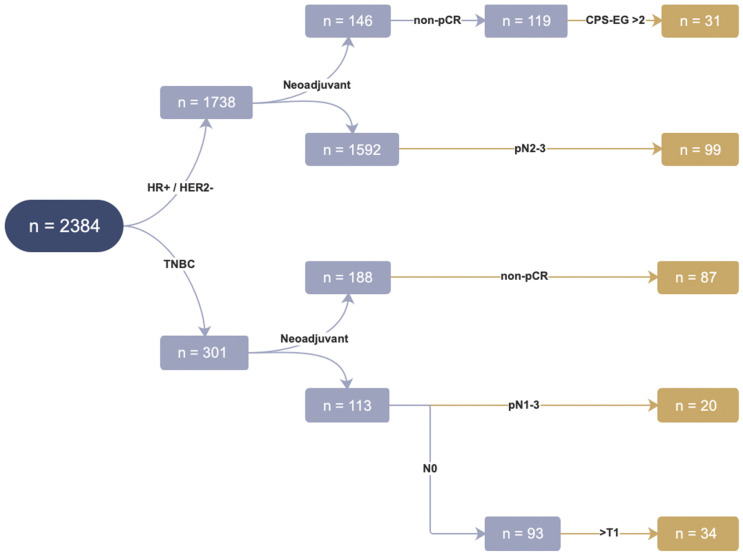
Patients fulfilling the OlympiA inclusion criteria: In total, 2384 patients comprised the study cohort, of whom 1738 patients were HR+/HER2− and 301 patients were triple-negative. A total of 146 of 1738 HR+/HER2− patients were treated with neoadjuvant chemotherapy. Of these, 119 patients displayed residual invasive tumor after surgery (non-pCR), and 31 of 119 patients with non-pCR exhibited a CPS-EG score > 2. In total, 1592 patients were either treated with adjuvant chemotherapy or no chemotherapy at all. Of these, 99 showed pathologic involvement of at least 4 lymph nodes (pN2-3). Of 301 patients with TNBC, 188 patients were treated with neoadjuvant chemotherapy and 87 of these patients had no pCR. A total of 113 of 301 patients were treated with adjuvant chemotherapy or no chemotherapy at all. Of these, 20 patients showed pathologic lymph node involvement (pN1-3), and 34 were node-negative but with a tumor size of at least 20 mm. In total, 130 of 1738 patients with HR+/HER2− breast cancer, 141 of 301 with TNBC and 271 of 2039 of all HER2−patients fulfilled the clinicopathological inclusion criteria of the OlympiA trial (displayed in brown).

In total, 271 of 2039 HER2− patients (13.3%) fulfilled the inclusion criteria of the OlympiA trial for clinical high risk (Figure 1). These included 130 of 1738 (7.5%) patients with HR+/HER2− breast cancer and 141 of 301 (46.8%) patients with TNBC (Figure 1).

Table 2 displays the characteristics of 271 patients who fulfilled the clinical high-risk criteria according to the OlympiA trial. Patients with HR+/HER2− tumor biology had an average age of 59.0 ± 14.1 years, and most of these patients were postmenopausal (90/130; 69.2%). Most HR+/HER2− patients had tumors larger than 20 mm (104/130; 80.0%) and at least four pathologically involved lymph nodes (111/130; 85.4%). Most patients with high-risk HR+/HER2− eBC displayed HER2 low receptor status (90/130; 69.2%). Approximately half of all clinical high-risk HR+/HER2− eBC patients showed a high Ki67 proliferation index (64/130; 49.2%), and most patients received chemotherapy (31/130 neoadjuvant, 23.8%; 56/130 adjuvant, 43.1%). A total of 34/130 (26.2%) patients with HR+/HER2− eBC met the criteria for hereditary cancer testing. Patients with TNBC were on average 57.9 ± 15.2 years old. Most of these patients were postmenopausal (81/141; 57.5%), and the most common grading was G3 (111/130; 78.7%). Regarding the TNBC patients, 52.5% (74/141) displayed a tumor size smaller than 20 mm, and 65.3% (92/141) did not have pathologically involved lymph nodes. Most patients with high-risk TNBC displayed HER2 0 receptor status (86/141; 61.0%). Of these patients, 92.2% (130/141) displayed a high Ki67 proliferative index, and 85.8% (121/141) of patients received chemotherapy (61.7% neoadjuvant, 87/141; and 24.1% adjuvant, 34/141). A total of 87/141 (61.7%) patients with TNBC met the criteria for hereditary cancer testing.

## 4. Discussion

The PARP inhibitor olaparib is the first of its kind to significantly prolong overall survival (OS) and invasive disease-free survival (IDFS) in patients with high-risk HR+/HER2− and triple-negative eBC who carry a germline BRCA1/2 mutation. We showed that nearly 8% of the patients with HR+/HER2− eBC and 47% of patients with early TNBC are potential candidates for olaparib treatment based on clinicopathologic risk factors and should therefore undergo gBRCA1/2 mutation testing. If gBRCA1/2 mutation testing was carried out based only on family history, the majority of patients (55% overall; 74% HR+/HER2−; 38% TNBC) potentially eligible for olaparib in our cohort would not receive gBRCA1/2 mutation testing.

As the test criteria of the GC-HBOC are similar to those of the NCCN and ESMO, our retrospective bicentric real-world data analysis from two large German breast centers is, in our opinion, not only representative of Germany but also of other western countries [11,12,13]. Nevertheless, the patient cohort characterized in this analysis differs from the patient cohort analyzed in the OlympiA trial, especially with regard to the use of chemotherapy. Patients could be enrolled in the OlympiA trial only if they had received neoadjuvant or adjuvant chemotherapy [5]. In our real-world analysis, approximately one third of all patients with clinically high-risk HR+/HER2− eBC, and approximately one seventh of the patients with TNBC, did not receive chemotherapy. Albeit suffering from breast cancer with a high risk of relapse or disease progression, and therefore presenting with a clear indication for (neo)adjuvant chemotherapy, chemotherapy might have been omitted due to patient choice, age or comorbidities. Moreover, other prognostic factors like (dynamic) Ki67 and multigene expression arrays impact chemotherapy decisions in HR+/HER2− early breast cancer [14,15,16,17,18,19].

The prevalence of gBRCA1/2 mutation depends on tumor biology, disease stage, sex, age, family predisposition, country and ethnicity [20]. In early TNBC, prevalence rates of gBRCA1/2 mutation range from 6.5%–15.4% [21,22,23]. In contrast, in HR+ eBC, the prevalence of gBRCA1/2 mutations range between 1.5% and 5.0% [21,24]. Taking these percent ranges into account, approximately 9–22 of 141 patients with high-risk TNBC, and 2–7 of 130 patients with high-risk HR+/HER2− eBC, would be gBRCA1/2 positive, would fulfill the clinicopathological inclusion criteria of OlympiA and would therefore benefit from adjuvant olaparib.

Patients with TNBC are most commonly treated with neoadjuvant chemotherapy. However, in our analysis, only 62.4% of the patients with TNBC received neoadjuvant treatment. As olaparib is an important treatment option for TNBC patients who carry gBRCA1/2 mutations and do not achieve a pCR after neoadjuvant chemotherapy, even more patients might have been eligible for adjuvant olaparib if all patients had received neoadjuvant chemotherapy. Other treatment options are available for patients who do not achieve a pCR [25]: according to the CREATE-X trial, postneoadjuvant capecitabine improves OS in patents with TNBC [26]. Moreover, the KEYNOTE-522 trial found that patients without a pCR will also benefit from the checkpoint inhibitor pembrolizumab. Although there are to date no clear data on which of these treatment options is the most effective for patients with TNBC and non-pCR, 64% of Expert Panel members at the St. Gallen Breast Cancer Consensus Conference 2021 voted to continue (postneo)adjuvant treatment with olaparib in patients with gBRCA-associated TNBC and residual tumor after neoadjuvant chemotherapy [27].

In HR+/HER2− eBC, nearly all patients who are eligible for adjuvant olaparib treatment also fulfill the inclusion criteria of the monarchE trial and are therefore candidates for a CDK4/6 inhibitor therapy with abemaciclib [2,28]. Moreover, ribociclib might be approved for use in high-risk HR+/HER2− eBC patients based on the findings of the phase III NATALEE trial, the first results of which were recently presented at the ASCO meeting [29,30]. There are, however, no data on whether olaparib or abemaciclib is more effective in these patients. At the St. Gallen International Breast Cancer Conference in 2023, 49% of Expert Panel members said that they would use both treatments in sequence. However, this decision rarely has to be made in clinical practice because, as discussed above, only few HR+/HER2− patients are eligible for treatment with olaparib. All patients that might receive both treatments in sequence should be informed about the off-label use, since no data are available for this treatment situation. Furthermore, all patients should be included in observational studies to assess potential short- and long-term side effects and potential cumulative toxicities of a sequential therapy with olaparib and a CDK4/6 inhibitor.

An important limitation of the current analysis is that, due to its retrospective nature, no information on the gBRCA1/2 mutation status was available. Yet, as indicated earlier, half of all patients in our cohort who displayed clinical high risk in line with the OlympiA trial criteria would not receive gBRCA1/2 mutation testing, according to the current criteria for hereditary cancer [31]. A recent study conducted by Yadav et al. found that BRCA1/2 mutation testing that is solely based on the NCCN guidelines would miss approximately 13% of patients with pathogenic gBRCA1/2 mutation [12,32]. Hence, all patients who are possibly eligible for olaparib treatment based on the clinicopathological high risk factors outlined in the OlympiA trial should undergo gBRCA1/2 testing [27].

## 5. Conclusions

In total, 8% of HR+/HER2− eBC and 47% of early TNBC patients display clinical high risk according to the OlympiA trial. As only 45% of these patients receive hereditary cancer testing according to the criteria of the GC-HBOC, a relevant proportion of gBRCA1/2-positive patients that might be eligible for olaparib treatment would be missed. Hence, all patients with eBC who fulfill the clinicopathologic eligibility criteria of the OlympiA trial should undergo gBRCA1/2 testing, irrespective of their own and family cancer history. Nevertheless, the total number of patients with early breast cancer who benefit from olaparib is estimated to be low. The true frequency of gBRA1/2 mutations in patients with eBC and clinical high risk, according to the OlympiA inclusion criteria, should be investigated in future studies.

## Figures and Tables

**Table 1:** Characteristics of HR+/HER2− and TNBC patients.

	HR+/HER2−	Percentage	TNBC	Percentage
**n**	1738	100	301	100
**Age**	60.1 ± 12.3		55.6 ± 14.9	
**Menopausal status**				
Premenopausal	491	28.2	129	42.9
Postmenopausal	1203	69.2	162	53.8
Male	3	0.2	0	0
n/a	41	2.4	10	3.3
**Histology**				
NST	1360	78.2	275	91.4
ILC	277	15.9	6	2.0
Other	100	5.8	18	6.0
n/a	1	0.1	2	0.6
**Grading**				
1	214	12.3	0	0
2	1247	71.8	59	19.6
3	275	15.8	238	79.1
n/a	2	0.1	4	1.3
T-stage *				
0	43	2.4	108	35.9
1	1041	59.9	126	41.9
2	540	31.1	52	17.3
3	80	4.6	10	3.3
4	34	2.0	5	1.6
N-stage *				
0	1195	68.8	252	83.7
1	415	23.9	36	12.0
2	89	5.1	8	2.7
3	38	2.1	5	1.6
X	1	0.1	0	0
**ER status**				
+	1729	99.5	0	0
−	9	0.5	301	100.0
**PR status**				
+	1447	83.3	0	0
−	291	16.7	301	100.0
**HER2 status**				
Positive	0	0.00	0	0
Low	1118	64.3	119	39.5
0	620	35.7	182	60.5
**Ki67**				
≥20%	580	33.4	279	92.7
<20%	1158	66.6	22	7.3
**Chemotherapy**				
Neoadjuvant	146	8.4	188	62.4
Adjuvant	313	18.0	72	23.9
None	1279	73.6	41	13.6

* T and N stages were assessed after surgery. NST, non-special type; ILC, invasive lobular carcinoma; ER, estrogen receptor; PR, progesterone receptor; HER2, human epidermal growth factor receptor 2; TNBC, triple-negative breast cancer; n/a, not available. Headlines are written in bold.

**Table 2:** Characteristics of patients that might be eligible for olaparib.

	HR+/HER2−	Percentage	TNBC	Percentage
**n**	130	100	141	100
**Age**	59.0 ± 14.1		57.9 ± 15.2	
**Menopausal status**				
Premenopausal	37	28.5	57	40.4
Postmenopausal	90	69.2	81	57.5
Male	0	0	0	0
n/a	3	2.3	3	2.1
**Histology**				
NST	105	80.8	121	85.8
ILC	21	16.2	4	2.8
Other	4	3.0	15	10.6
n/a	0	0	1	0.8
**Grading**				
1	0	0	0	0
2	90	69.2	30	21.3
3	40	30.8	111	78.7
n/a	0	0	0	0
T-stage *				
0	3	2.3	7	5.0
1	23	17.7	67	47.5
2	72	55.4	52	36.9
3	20	15.4	10	7.1
4	12	9.2	5	3.5
N-stage *				
0	4	3.1	92	65.3
1	15	11.5	36	25.5
2	73	56.2	8	5.7
3	38	29.2	5	3.5
X	0	0	0	0
**ER status**				
+	126	96.9	0	0
-	4	3.1	141	100.0
**PR status**				
+	97	74.6	0	0
-	33	25.4	141	100.0
**HER2 status**				
Positive	0	0	0	0
Low	90	69.2	55	39.0
0	40	30.8	86	61.0
**Ki67**				
≥20%	64	49.2	130	92.2
<20%	66	50.8	11	7.8
**Chemotherapy**				
Neoadjuvant	31	23.8	87	61.7
Adjuvant	56	43.1	34	24.1
None	43	33.1	20	14.2
**Criteria for hereditary cancer testing met**				
no	96	73.8	54	38.3
yes	34	26.2	87	61.7

* T and N stages were assessed after surgery. NST, non-special type; ILC, invasive lobular carcinoma; ER, estrogen receptor; PR, progesterone receptor; HER2, human epidermal growth factor receptor 2; TNBC, triple-negative breast cancer; n/a, not available. Headlines are written in bold.

## Data Availability

The data presented in this study are available on request from the corresponding author. The data are not publicly available for the privacy of sensitive patient information.

## Supplementary Materials

The following supporting information can be downloaded at: https://www.mdpi.com/article/10.3390/cancers15153847/s1, Table S1: Characteristics of the total patient cohort, Table S2: Indication for genetic testing in the BRCA1/2 genes and potentially other risk genes [11].

## References

[1] Harbeck N., Rastogi P., Martin M., Tolaney S.M., Shao Z.M., Fasching P.A., Huang C.S., Jaliffe G.G., Tryakin A., Goetz M.P. (2021). Adjuvant abemaciclib combined with endocrine therapy for high-risk early breast cancer: Updated efficacy and Ki-67 analysis from the monarchE study. Ann. Oncol..

[2] Johnston S.R.D., Harbeck N., Hegg R., Toi M., Martin M., Shao Z.M., Zhang Q.Y., Martinez Rodriguez J.L., Campone M., Hamilton E. (2020). Abemaciclib Combined With Endocrine Therapy for the Adjuvant Treatment of HR+, HER2−, Node-Positive, High-Risk, Early Breast Cancer (monarchE). J. Clin. Oncol..

[3] Johnston S.R.D., Toi M., O’Shaughnessy J., Rastogi P., Campone M., Neven P., Huang C.S., Huober J., Jaliffe G.G., Cicin I. (2023). Abemaciclib plus endocrine therapy for hormone receptor-positive, HER2-negative, node-positive, high-risk early breast cancer (monarchE): Results from a preplanned interim analysis of a randomised, open-label, phase 3 trial. Lancet Oncol..

[4] Chan A., Delaloge S., Holmes F.A., Moy B., Iwata H., Harvey V.J., Robert N.J., Silovski T., Gokmen E., von Minckwitz G. (2016). Neratinib after trastuzumab-based adjuvant therapy in patients with HER2-positive breast cancer (ExteNET): A multicentre, randomised, double-blind, placebo-controlled, phase 3 trial. Lancet Oncol..

[5] Tutt A.N.J., Garber J.E., Kaufman B., Viale G., Fumagalli D., Rastogi P., Gelber R.D., de Azambuja E., Fielding A., Balmaña J. (2021). Adjuvant Olaparib for Patients with BRCA1- or BRCA2-Mutated Breast Cancer. N. Engl. J. Med..

[6] Geyer C.E., Garber J.E., Gelber R.D., Yothers G., Taboada M., Ross L., Rastogi P., Cui K., Arahmani A., Aktan G. (2022). Overall survival in the OlympiA phase III trial of adjuvant olaparib in patients with germline pathogenic variants in BRCA1/2 and high risk, early breast cancer. Ann. Oncol..

[7] Mittendorf E.A., Jeruss J.S., Tucker S.L., Kolli A., Newman L.A., Gonzalez-Angulo A.M., Buchholz T.A., Sahin A.A., Cormier J.N., Buzdar A.U. (2011). Validation of a novel staging system for disease-specific survival in patients with breast cancer treated with neoadjuvant chemotherapy. J. Clin. Oncol..

[8] Kast K., Rhiem K., Wappenschmidt B., Hahnen E., Hauke J., Bluemcke B., Zarghooni V., Herold N., Ditsch N., Kiechle M. (2016). Prevalence of BRCA1/2 germline mutations in 21 401 families with breast and ovarian cancer. J. Med. Genet..

[9] Engel C., Rhiem K., Hahnen E., Loibl S., Weber K.E., Seiler S., Zachariae S., Hauke J., Wappenschmidt B., Waha A. (2018). Prevalence of pathogenic BRCA1/2 germline mutations among 802 women with unilateral triple-negative breast cancer without family cancer history. BMC Cancer.

[10] Harter P., Hauke J., Heitz F., Reuss A., Kommoss S., Marmé F., Heimbach A., Prieske K., Richters L., Burges A. (2017). Prevalence of deleterious germline variants in risk genes including BRCA1/2 in consecutive ovarian cancer patients (AGO-TR-1). PLoS ONE.

[11] GCHBOC Indications for Genetic Testing. https://www.konsortium-familiaerer-brustkrebs.de/betreuungskonzept/molekulare-diagnostik/indikationen-gentest/.

[12] Daly M., Pal T., AlHilli Z. NCCN Clinical Practice Guidelines in Oncology: Genetic/Familial High-Risk Assessment: Breast, Ovarian, and Pancreatic. https://www.nccn.org.

[13] Sessa C., Balmaña J., Bober S., Cardoso M.-J., Colombo N., Curigliano G., Domchek S., Evans D., Fischerova D., Harbeck N. (2023). Risk reduction and screening of cancer in hereditary breast-ovarian cancer syndromes: ESMO Clinical Practice Guideline. Ann. Oncol..

[14] Cardoso F., van’t Veer L.J., Bogaerts J., Slaets L., Viale G., Delaloge S., Pierga J.Y., Brain E., Causeret S., DeLorenzi M. (2016). 70-Gene Signature as an Aid to Treatment Decisions in Early-Stage Breast Cancer. N. Engl. J. Med..

[15] Sparano J.A., Gray R.J., Makower D.F., Pritchard K.I., Albain K.S., Hayes D.F., Geyer C.E., Dees E.C., Goetz M.P., Olson J.A. (2018). Adjuvant Chemotherapy Guided by a 21-Gene Expression Assay in Breast Cancer. N. Engl. J. Med..

[16] Kalinsky K., Barlow W.E., Gralow J.R., Meric-Bernstam F., Albain K.S., Hayes D.F., Lin N.U., Perez E.A., Goldstein L.J., Chia S.K.L. (2021). 21-Gene Assay to Inform Chemotherapy Benefit in Node-Positive Breast Cancer. N. Engl. J. Med..

[17] Dubsky P., Filipits M., Jakesz R., Rudas M., Singer C.F., Greil R., Dietze O., Luisser I., Klug E., Sedivy R. (2013). EndoPredict improves the prognostic classification derived from common clinical guidelines in ER-positive, HER2-negative early breast cancer. Ann. Oncol..

[18] Dannehl D., Engler T., Volmer L.L., Staebler A., Fischer A.K., Weiss M., Hahn M., Walter C.B., Grischke E.M., Fend F. (2022). Recurrence Score(^®^) Result Impacts Treatment Decisions in Hormone Receptor-Positive, HER2-Negative Patients with Early Breast Cancer in a Real-World Setting-Results of the IRMA Trial. Cancers.

[19] Tolaney S.M., Guo H., Pernas S., Barry W.T., Dillon D.A., Ritterhouse L., Schneider B.P., Shen F., Fuhrman K., Baltay M. (2019). Seven-Year Follow-Up Analysis of Adjuvant Paclitaxel and Trastuzumab Trial for Node-Negative, Human Epidermal Growth Factor Receptor 2-Positive Breast Cancer. J. Clin. Oncol..

[20] Armstrong N., Ryder S., Forbes C., Ross J., Quek R.G. (2019). A systematic review of the international prevalence of BRCA mutation in breast cancer. Clin. Epidemiol..

[21] Hu C., Hart S.N., Gnanaolivu R., Huang H., Lee K.Y., Na J., Gao C., Lilyquist J., Yadav S., Boddicker N.J. (2021). A Population-Based Study of Genes Previously Implicated in Breast Cancer. N. Engl. J. Med..

[22] Wong-Brown M.W., Meldrum C.J., Carpenter J.E., Clarke C.L., Narod S.A., Jakubowska A., Rudnicka H., Lubinski J., Scott R.J. (2015). Prevalence of BRCA1 and BRCA2 germline mutations in patients with triple-negative breast cancer. Breast Cancer Res. Treat..

[23] Sharma P., Klemp J.R., Kimler B.F., Mahnken J.D., Geier L.J., Khan Q.J., Elia M., Connor C.S., McGinness M.K., Mammen J.M. (2014). Germline BRCA mutation evaluation in a prospective triple-negative breast cancer registry: Implications for hereditary breast and/or ovarian cancer syndrome testing. Breast Cancer Res. Treat..

[24] Tung N., Lin N.U., Kidd J., Allen B.A., Singh N., Wenstrup R.J., Hartman A.-R., Winer E.P., Garber J.E. (2016). Frequency of germline mutations in 25 cancer susceptibility genes in a sequential series of patients with breast cancer. J. Clin. Oncol..

[25] Trapani D., Ferraro E., Giugliano F., Boscolo Bielo L., Curigliano G., Burstein H.J. (2022). Postneoadjuvant treatment for triple-negative breast cancer. Curr. Opin. Oncol..

[26] Masuda N., Lee S.-J., Ohtani S., Im Y.-H., Lee E.-S., Yokota I., Kuroi K., Im S.-A., Park B.-W., Kim S.-B. (2017). Adjuvant capecitabine for breast cancer after preoperative chemotherapy. N. Engl. J. Med..

[27] Burstein H.J., Curigliano G., Thürlimann B., Weber W.P., Poortmans P., Regan M.M., Senn H.J., Winer E.P., Gnant M. (2021). Customizing local and systemic therapies for women with early breast cancer: The St. Gallen International Consensus Guidelines for treatment of early breast cancer 2021. Ann. Oncol..

[28] Dannehl D., Volmer L.L., Weiss M., Matovina S., Grischke E.M., Oberlechner E., Seller A., Walter C.B., Hahn M., Engler T. (2022). Feasibility of Adjuvant Treatment with Abemaciclib-Real-World Data from a Large German Breast Center. J. Pers. Med..

[29] Slamon D.J., Fasching P.A., Patel R., Verma S., Hurvitz S.A., Chia S.K.L., Crown J., Martin M., Barrios C.H., Spera G. (2019). NATALEE: Phase III study of ribociclib (RIBO) + endocrine therapy (ET) as adjuvant treatment in hormone receptor–positive (HR+), human epidermal growth factor receptor 2–negative (HER2–) early breast cancer (EBC). J. Clin. Oncol..

[30] Stroyakovskiy D., Yardley D.A., Huang C.-S., Fasching P.A., Crown J., Bardia A., Chia S., Im S.-A., Martin M., Loi S. (2023). Ribociclib and endocrine therapy as adjuvant treatment in patients with HR+/HER2− early breast cancer: Primary results from the phase III NATALEE trial. J. Clin. Oncol..

[31] Rhiem K., Auber B., Briest S., Dikow N., Ditsch N., Dragicevic N., Grill S., Hahnen E., Horvath J., Jaeger B. (2022). Consensus recommendations of the German Consortium for hereditary breast and ovarian cancer. Breast Care.

[32] Yadav S., Hu C., Hart S.N., Boddicker N., Polley E.C., Na J., Gnanaolivu R., Lee K.Y., Lindstrom T., Armasu S. (2020). Evaluation of Germline Genetic Testing Criteria in a Hospital-Based Series of Women With Breast Cancer. J. Clin. Oncol..

