# Supplementary material for: Which Patients Do We Need to Test for BRCA1/2 Mutation? Feasibility of Adjuvant Olaparib Treatment in Early Breast Cancer–Real-World Data from Two Large German Breast Centers
Source: Cancers (Basel). 2023 Jul 28;15(15):3847. doi: 10.3390/cancers15153847 (PMC10417328; doi:10.3390/cancers15153847)
Supplement: Supplementary file 1 [file cancers-15-03847-s001.zip › cancers-2490325-supplementary.pdf]

cancers

MDPI

Table S1. Characteristics of the total patient cohort.

		Percentage
**n**	2384	100
**Age**	59.0 ± 12.8	
**Menopausal status**		
Premenopausal	750	31.5
Postmenopausal	1575	66.1
Male	3	0.1
n/a	56	2.3
**Histology**		
NST	1951	81.8
ILC	301	12.6
Other	127	5.4
n/a	5	0.2
**Grading**		
1	214	9.0
2	1470	61.7
3	694	29.1
n/a	6	0.2
**T-stage** *	0	
0	272	11.4
1	1315	55.2
2	657	27.6
3	97	4.0
4	43	1.8
**N-stage** *		
0	1712	71.8
1	513	21.5
2	112	4.7
3	46	1.9
X	1	0.1
**ER status**		
+	1971	82.7
−	413	17.3
**PR status**		
+	1614	67.7
−	770	32.3
**HER2 status**		
Positive	345	14.4
Low	1234	51.8
0	**805**	33.8
**Ki67**		
≥20 %	1126	47.2
<20 %	1258	52.8
**Chemotherapy**		
Neoadjuvant	505	21.2
Adjuvant	507	21.3
None	1372	57.5

* T and N stages were assessed after surgery. NST, non-special type; ILC, invasive lobular carcinoma; ER, estrogen receptor; PR, progesterone receptor; HER2, human epidermal growth factor receptor 2; TNBC, triple-negative breast cancer; n/a, not available. Headlines are written in bold.

Table S2. Indication for genetic testing in the BRCA1/2 genes and potentially other risk genes:

**Families with a minimum of: (Regarding one side of the family)**
- Three women affected by breast cancer, regardless of age.<br>– Two women affected by breast cancer, with one diagnosis occurring before the age of 51.<br>– One woman affected by breast cancer and one woman affected by ovarian cancer<br>– One woman affected by both breast and ovarian cancer<br>– Two women affected by ovarian cancer<br>– One woman affected by bilateral breast cancer, with one diagnosis occurring before the age of 51<br>– One woman affected by breast cancer before the age of 36<br>– One man affected by breast cancer
Further criteria:
- Personal history of triple-negative breast cancer diagnosed before the age of 60<br>- Personal history of ovarian cancer diagnosed before the age of 80

Translated from German to English, comparable to the German version found at [11].
